# Outcomes of cystoid macular edema following Descemet’s membrane endothelial keratoplasty in a referral center for keratoplasty in Spain: retrospective study

**DOI:** 10.1038/s41598-023-29127-5

**Published:** 2023-02-09

**Authors:** Nuno Moura-Coelho, Renato Papa-Vettorazzi, Imalvet Santiesteban-García, Arnaldo Dias-Santos, Felicidad Manero, João Paulo Cunha, José Güell

**Affiliations:** 1 Cornea and Refractive Surgery Unit, Instituto Microcirurgia Ocular (IMO), Carrer Josep Maria Lladó, 3, 08035 Barcelona, Spain; 2 NOVA Medical School, Faculdade de Ciências Médicas – Universidade Nova de Lisboa (NMS|FCM-UNL), Lisbon, Portugal; 3 European School for Advanced Studies in Ophthalmology (ESASO), Lugano, Switzerland; 4 Ophthalmology, Hospital CUF Cascais, Cascais, Portugal; 5 Department of Ophthalmology, Hospital CUF Descobertas, Lisbon, Portugal; 6 Ophthalmology, Centro Hospitalar Universitário Lisboa Central (CHULC), Lisbon, Portugal; 7 Universidad Autónoma de Barcelona (UAB), Barcelona, Spain; 8 Escola Superior de Tecnologias da Saúde de Lisboa (ESTeSL) – Instituto Politécnico de Lisboa, Lisbon, Portugal

**Keywords:** Pathogenesis, Risk factors, Signs and symptoms

## Abstract

The aim of this study was to analyze the outcomes of eyes with visually significant cystoid macular œdema (vs-CMO) after Descemet membrane endothelial keratoplasty (DMEK) in a referral center for keratoplasty in Spain. We conducted a retrospective, single-surgeon case series of eyes that developed post-DMEK vs-CMO performed between January 2011 and December 2020. Data collected included: indication for DMEK; biometric data; ocular comorbidities; past medical history; time to detection of vs-CMO after DMEK (T, weeks); best-corrected visual acuity (BCVA, logMAR) and central retinal thickness (CRT, µm) at diagnosis of vs-CMO, after resolution of CMO, and at last follow-up; and management strategy. Main outcomes analyzed were incidence of vs-CMO, improvement in BCVA and CRT after treatment of vs-CMO. Of 291 consecutive DMEK surgeries, 14 eyes of 13 patients (4.8%) developed vs-CMO. Five patients (38.5%) had history of CMO, and 28.6% of eyes had ophthalmic comorbidities. Median (P25-P75) T was 4 (3–10) weeks. Treatment success was observed in 12/13 eyes (92.3%), two of which required second-line treatment. In successful cases (median time-to-resolution 3.0 (2.0–3.5) months), median BCVA improved from 0.60 (0.40–0.80) logMAR to 0.30 (0.15–0.40) logMAR (p = 0.002) after treatment, and median CRT improved from 582.5 (400.0–655.0) µm to 278.0 (258.0–294.0) µm (p = 0.005). In our study, we found a 4.8% rate of post-DMEK vs-CMO, with most cases occurring in the first 3 months after surgery. Good functional and anatomical outcomes are expected in most eyes, without treatment-related complications or implications in graft outcomes. Additional studies are encouraged to determine a standardized protocol for post-DMEK vs-CMO.

## Introduction

Endothelial keratoplasty (EK) techniques have become the gold standard for the treatment of corneal endothelial failure, given the better visual outcomes, faster visual recovery, more predictable refractive error and astigmatism, and lower complication rates compared with penetrating keratoplasty (PKP)^1^.

Postoperative cystoid macular oedema (CMO) is the most frequent posterior segment complication reported after EK^2,3^, including both Descemet stripping endothelial keratoplasty (DSEK) and Descemet membrane endothelial keratoplasty (DMEK), but even so the incidence of CMO after EK compares favourably with its incidence after PKP^4^.

Importantly, most studies of CMO after DMEK have focused on detection of CMO at postoperative spectral-domain optical coherence tomography scans (SD-OCT), but to the best of the authors’ knowledge few studies have focused on the outcomes of “visually significant cystoid macular oedema” (vs-CMO)^4–7^. Treatment of post-DMEK CMO is usually favourable, but different first-line treatments have been proposed and few reports are available^3,5,8,9^.

The aim of this study was to assess the proportion of eyes that developed vs-CMO following DMEK, and to analyse the anatomical and visual outcomes of post-DMEK vs-CMO in a referral centre for DMEK in Spain.

## Materials and methods

### Study design and approval

This was a retrospective, observational case series of eyes that developed vs-CMO after DMEK. All patients signed an institutional review board-approved informed consent. The study was conducted according to the tenets of the Declaration of Helsinki.

Visually significant CMO after DMEK was defined as:

- The presence of intra- or sub-retinal fluid in the foveal region detected by macular SD-OCT scans in patients where CMO was suspected;
- Cystoid macular edema was suspected on the basis of a lower-than-expected postoperative visual acuity: despite a clear cornea after DMEK and/or the absence of abnormal de novo funduscopic findings”^5,6^.

### Surgical technique and postoperative protocol

Pre-cut corneal donor tissues for primary DMEK grafts were obtained from the Barcelona Tissue Eye Bank. DMEK surgeries were all performed by a single surgeon (J.G.).The surgical technique for primary DMEK surgery was as previously described by our group^10,11^. DMEK graft positioning onto the recipient posterior stroma was performed using 20% sulfur hexafluoride (SF6) as tamponade. Subconjunctival dexamethasone 80 mg was administered at the end of the surgery. To ensure graft attachment with the aid of SF 20% bubble, patients were instructed on different positioning regimens in the early postoperative period, as we have previously described^10,11^.

Postoperative treatment was as we previously described^12,13^, and consisted of topical tobramycin 0.3% and dexamethasone 0.1% (Tobradex; Alcon Cusi, El Mas Nou, Barcelona, Spain) every 2 h for the first postoperative day, then 6 times daily for one week, then 4 times daily for 4 weeks and then tapering over the following 3 months (reduction in 1 drop every 4 weeks); timolol 0.5% (Cusimolol; Alcon Cusi) eye drops 2 times daily for 12 weeks, with additional ocular hypotensive medications if needed; and dexamethasone 0.05% and chloramphenicol 1% ointment at nighttime (DeIcol; Alcon Cusi) for 6 months. Oral methylprednisolone (Urbason; Sanofi Aventis Pharma SA, Barcelona, Spain) was also prescribed and slowly tapered off for the first 3 weeks: 40 mg/day for 3 days; 20 mg/day for 3 more days; 10 mg/day for 1 week; and 10 mg every 48 h for 1 week. A topical corticosteroid was kept at least once daily indefinitely after DMEK, unless contraindicated in light of significant increases in intraocular pressure.

In patients with vs-CMO after DMEK, first-line treatment consisted of adding a topical non-steroidal anti-inflammatory (NSAID) drug twice daily (either bromfenac 0.9 mg/mL (Yellox, Bausch & Lomb) or nepafenac 0.1 mg/mL (Nevanac, Novartis)) to the topical steroid regime plus oral acetazolamide 250 mg three times per day (plus oral potassium supplementation) until resolution of CMO. In cases of incomplete response to first-line treatment, the second-line treatment consisted of intravitreal injection of corticosteroids.

### Data collection

We analyzed the patients’ electronic medical records (EMRs) to identify cases where a diagnosis of vs-CMO after DMEK was made. The following data was collected retrospectively from the patients’ EMRs: demographic data (age, gender, laterality); indication for DMEK surgery; relevant past medical and ocular history (based on evidence from studies of pseudophakic CMO^14–16^ and from previous studies on risk factors for postoperative CMO after DSAEK^17,18^), including diabetes mellitus, glaucoma, retinal disease, uveitis, history of CMO in either the interest eye or in the fellow eye; surgical procedure (whether standalone DMEK or DMEK combined with other intraocular procedures); re-bubbling for partial or complete graft detachment; interval between DMEK and detection of vs-CMO (T, weeks); best-corrected visual acuity (BCVA, logMAR) before DMEK, at time of detection of vs-CMO, at resolution of vs-CMO, and at final follow-up observation (F-U); response to first-line treatments and subsequent treatments performed for vs-CMO; resolution of vs-CMO and time to resolution (months); central retinal thickness (CRT, µm) as measured by SD-OCT in the central ETDRS circle at detection of vs-CMO and at last F-U; other postoperative complications, including intraocular pressure (IOP) spikes, ocular hypertension or glaucoma, IOL opacification or cataractogenesis, immune rejection episodes, and graft failure.

Primary outcomes analyzed included the proportion of eyes (%) developing vs-CMO during the first 6 months after DMEK, % eyes with resolved CMO, and change in BCVA after resolution of vs-CMO compared with BCVA before treatment for vs-CMO. Other outcomes analyzed were improvement in CRT after treatment for vs-CMO; % eyes reaching final BCVA ≤ 0.30 logMAR (≥ 20/40 Snellen), % eyes reaching final BCVA ≤ 0.10 logMAR (≥ 20/25 Snellen) and % eyes reaching final BCVA ≤ 0 logMAR (≥ 20/20 Snellen) at last F-U; and success rate of treatment.

### Statistical analysis

Data was collected from the patients’ EMRs to a database in Microsoft Excel^®^**,** and data was then exported to SPSS software (v 27.0; IBM Corp, Chicago, Illinois, USA) for statistical analysis. Quantitative variables were described as mean (standard deviation) if they had a normal distribution, and parametric tests were applied in this case; otherwise, variables without a normal distribution were reported as median (P25-P75), and nonparametric tests were applied. We considered a significance level α of 0.05.

### Compliance with ethical standards

The study received approval from the institutional Ethics and Research Committee (CEIm Institut de Microcirurgia Ocular—IMO). All patients signed an institutional review board-approved informed consent. The study was conducted according to the tenets of the Declaration of Helsinki.

## Results

### Baseline data

**Figure 1:**
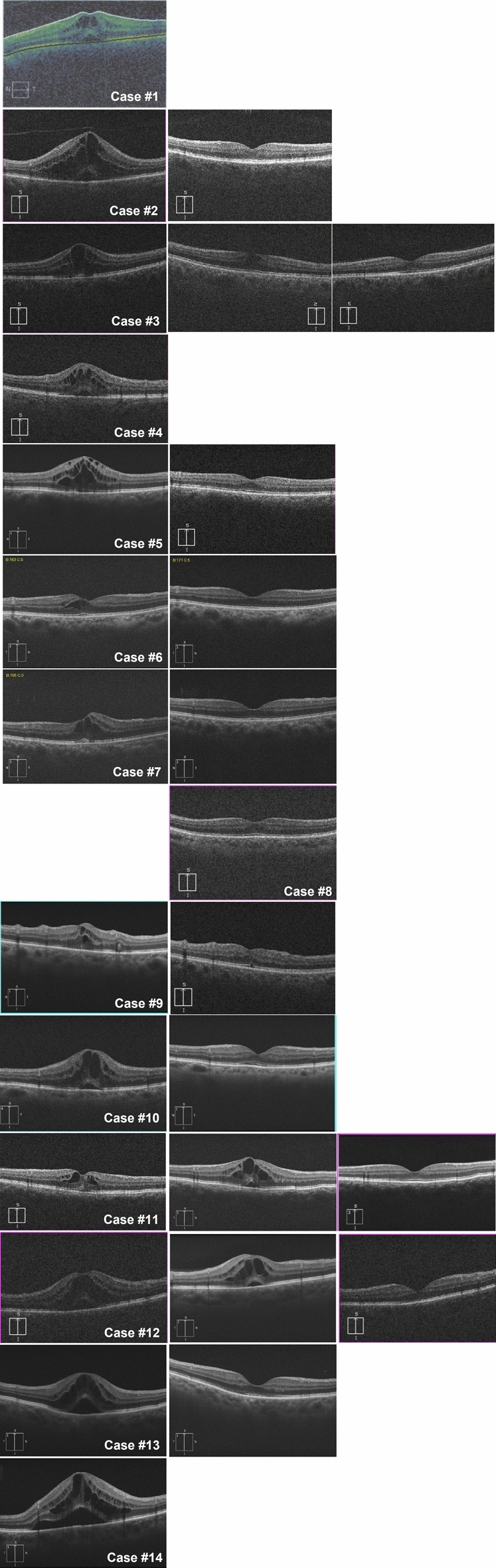
Macular spectral-domain optical coherence tomography (SD-OCT) images of the cases of postoperative visually significant cystoid macular oedema (vs-CMO). In Cases #3 and #12, SD-OCT scans at diagnosis of vs-CMO, before intravitreal corticosteroid injection, and after treatment are shown. In Case #11, CMO was present before DMEK, and worsening of macular oedema was observed after DMEK, improving after treatment. The SD-OCT image after treatment of vs-CMO was not available in the electronic medical records (EMR) of Case #1. Cases #4 and #14 had were lost to follow-up. Case #8 was diagnosed with vs-CMO, but the SD-OCT images were not available in the patient’s EMRs.

Demographic and baseline patient data are presented in Table 1; individual patient data is presented in Supplementary Table 1. Of 291 consecutive DMEK surgeries, fourteen eyes of 13 patients (69.2% female) developed vs-CMO after DMEK (4.8% incidence); one of the eyes had persistent macular oedema before DMEK,which worsened after DMEK surgery (Fig. 1, Case #11). Mean patient age at the time of DMEK surgery was 63, 4 (10.3) years. Two patients (2/13, 15.4%) had known history of diabetes mellitus. Indication for DMEK was Fuchs corneal endothelial dystrophy (FECD) in 7 eyes (50.0%), pseudophakic bullous keratopathy in three eyes (21.4%), redo DMEK in two eyes (14.3%), phakic intraocular lens-related corneal decompensation in one eye (7.1%), and angle-closure glaucoma-related endothelial failure in one eye (7.1%). Eight eyes had axial length (AL) data, with a mean AL of 23.08 (1.55) mm and seven eyes had anterior chamber depth (ACD) data, with a mean ACD of3.37 (0.92) mm; of note,four eyes (28.6%) had shallow anterior chambers preoperatively, with three of them having history of iridotomy and one of them having history of acute angle-closure glaucoma. Three patients (3/13, 23.1%) had history of DMEK in the fellow eye. Five patients (5/13, 38.5%) had history of postoperative CMO in either the surgical eye or in the fellow eye; one of these patients had persistent, partially refractory postoperative CMEat the time of DMEK surgery. Four eyes (28.6%) had some ophthalmic comorbidity limiting visual potential after DMEK (one eye had macular drusen and angle-closure glaucoma, one eye had an epiretinal membrane, one eye was amblyopic, and one eye had glaucoma). Three eyes (21.4%) had staged DMEK surgery, preceded by cataract surgery less than 4 months before DMEK; the remainder underwent standalone DMEK surgery or DMEK graft exchange. After DMEK, re-bubbling was performed in two eyes (2/14, 14.3%), one of which underwent two re-bubbling procedures.

**Table 1:** Patient demographics and DMEK perioperative data.

Patients’ characteristics N = 14 eyes (13 patients)	
Demographic data	
Age, years	63.4 (10.3)
Female gender, n (%)	9/13 (69.2%)
Right eyes, n (%)	8/14 (57.1%)
Biometry data	
Anterior chamber depth, mm (n = 7)	3.37 (0.92)*
Axial length, mm (n = 8)	23.08 (1.55)
Ophthalmic history	
Ocular comorbidities, n (%)	4 (28.6%)
	Dry mild ARMD and glaucoma (1), ERM (1), Glaucoma (1),Amblyopia (1)
Previous corneal grafts, n (%)**	5 (35.7%)
	Interest eye (2), Fellow eye (3)
History of postoperative CMO, n (%)**	5 (35.7%)
	Interest eye (3), Fellow eye (2)
DMEK data	
Indication for DMEK (n, %)	FECD (7, 50.0%)
	PPBK (3, 21.4%)
	Redo DMEK (2, 14.3%)
	Glaucoma-related corneal decompensation (1, 7.1%)
	PIOL-related corneal decompensation (1, 7.1%)
Surgical technique (n, %)	Standalone DMEK (11/14, 78.6%)***
	Staged DMEK surgery (3/14, 21.4%)
Re-bubbling after DMEK, n (%)	2 (14.3%)

Quantitative variables are presented as mean (SD) or as median (P25-P75), depending on whether the variable followed a normal distribution. Categorical variables are presented as absolute and relative frequencies (n, %).

*ARMD* age-related macular degeneration, *ERM* epiretinal membrane, *CMO* cystoid macular oedema, *DMEK* Descemet’s membrane endothelial keratoplasty, *FECD* Fuchs’ endothelial corneal dystrophy, *PPBK* pseudophakic bullous keratopathy, *PIOL* phakic intraocular lens.

*Four eyes (28.6%) had shallow anterior chambers preoperatively, with three of them having history of iridotomy, of which one had history of acute angle-closure glaucoma.

**Either in the interest eye or in the fellow eye.

***Two of the standalone DMEK surgeries were DMEK graft exchange.

In eyes without pre-existing CMO at the time of DMEK (n = 13), median time to detection of vs-CMO (T) was 4 (3–10) weeks; vs-CMO was detected during the first postoperative month in eight eyes (61.5%), and all cases vs-CMO were detected within the first 6 months after DMEK. Because patients referred for DMEK to our center are from a broad referral base, some patients performed SD-OCT elsewhere, and so OCT data before treatment was not available in our institution’s EMRs for one case and the OCT data after treatment was not available in the EMRs of another case. The SD-OCT data analysis was thus performed in the cases where macular SD-OCT scans were retrieved from the patients’ EMRs (Supplementary Table 1). In the cases where SD-OCT before and after treatment was retrieved from the patients’ EMRs (n = 10), all eyes had intraretinal fluid, and six eyes (6/10, 60.0%) had subretinal fluid. There were two eyes with loss to follow-up (LTFU): one eye was lost to follow-up after diagnosis and so was not included in the success of treatment analysis; the second had persistent CMO at M4 post-DMEK under first-line treatment and was then LTFU, which we considered as a case of treatment failure. These cases were excluded from the BCVA and CRT analysis, and visual outcomes were reported for twelve eyes with successfully resolved CMO after treatment (12/13 eyes, 92.3%).SD-OCT macular scans available in the patients’ EMRs are presented for all cases in Fig. 1.

### Visual acuity and central retinal thickness outcomes

In eyes with resolution of CMO after treatment (n = 12), first-line treatment was effective in ten eyes (83.3%). Two eyes (16.7%) required second-line treatments with intravitreal CS (sustained-release 0.7-mg dexamethasone implant (Ozurdex, Allergan) in one case, and intravitreal injection of triamcinolone acetonide 40 mg/mL (Trigon Depot, Bristol-Myers-Squib) in another case). Both cases had resolution of CMO with full visual and anatomical recovery; however, the second eye had a complicated clinical course with rejection at M8 and IOL opacification requiring IOL exchange plus anterior vitrectomy, and eventually secondary graft failure at M21 post-DMEK.

In eyes with resolution of CMO after treatment (n = 12), there was a statistically significant improvement in median BCVA, from 0.60 (0.40–0.80) logMAR at the time of detection of vs-CMO to 0.30 (0.15–0.40) logMAR (p = 0.002, Wilcoxon signed rank test). Median time to resolution of vs-CMO was 3.0 (2.0–3.5) months. In eyes without eventual graft failure (n = 10), there was a statistically significant improvement in BCVA at last F-U observation ( compared with baseline BCVA before DMEK surgery (0.02 (0.00–0.20) logMAR versus 0.58 (0.40–1.00) logMAR; p = 0.005, Wilcoxon signed rank test); all eyes reached final BCVA ≤ 0.30 logMAR (≥ 20/40 Snellen), 70% of eyes reached final BCVA ≤ 0.10 logMAR (≥ 20/25 Snellen), and 50% of eyes reached final BCVA ≤ 0 logMAR (≥ 20/20 Snellen).

CRT analysis was performed only in cases with SD-OCT images available before and after treatment of vs-CMO (n = 10) (Table 2). There was a statistically significant improvement in median CRT after treatment of vs-CMO, from 582.5 (400.0–655.0) µm at diagnosis of vs-CMO to 278.0 (258.0–294.0) µm (p = 0.005, Wilcoxon signed rank test).

**Table 2:** Visual outcomes and macular spectral-domain optical coherence tomography data after treatment of post-DMEK visually significant cystoid macular oedema.

Post-DMEK vs-CMO data and outcomes	
**BCVA, logMAR (n = 12)**	
BCVA before DMEK, logMAR (n = 12)	0.58 (0.35–0.85) logMAR
BCVA at diagnosis of vs-CMO, logMAR (n = 12)	0.60 (0.40–0.80) logMAR
BCVA after resolution of vs-CMO, logMAR (n = 12)	0.30 (0.15–0.40) logMAR
BCVA at last F-U, logMAR (n = 10)*	0.02 (0.00–0.20) logMAR
Macular SD-OCT data (n = 10)**	
CRT at diagnosis of post-DMEK vs-CMO, µm	582.5 (400.0-655.0) µm
Presence of IRF/SRF at diagnosis, n (%)	IRF 10 (100%)/SRF 6 (60%)
CRT after treatment vs-CMO, µm	278.0 (258.0–294.0) µm

Quantitative variables are presented as mean (SD) or as median (P25-P75), depending on whether the variable followed a normal distribution. Categorical variables are presented as absolute and relative frequencies (n, %).

*DMEK* Descemet’s membrane endothelial keratoplasty, *vs-CMO* visually significant cystoid macular oedema, *BCVA* best-corrected visual acuity, *F-U* follow-up, *SD-OCT* spectral-domain optical coherence tomography, *CRT* central retinal thickness, *IRF* intraretinal fluid, *SRF* subretinal fluid.

*BCVA analysis at last F-U excluding two eyes with graft failure.

**SD-OCT data statistical analysis was only performed in patients where OCT scans were available before and after treatment of cystoid macular oedema.

### Corneal clearing and DMEK graft survival

Of the eyes with resolution of CMO after treatment (n = 12), mean F-U time after DMEK surgery was 66.3 (32.0) months (range 18–126 months). At last F-U, ten eyes (10/12, 83.3%) had a clear cornea and a functioning DMEK graft. One eye (7.6%) had a complicated course after resolution of vs-CMO, with rejection at M8 and IOL exchange due to IOL opacification, with secondary graft failure 21 months after DMEK surgery. The other case had an immune rejection episode at 34-month follow-up, and graft failure was observed 66 months after DMEK.

## Discussion

This is the first study to characterize the incidence and outcomes of postoperative vs-CMO after DMEK in the Iberic Peninsula. In our study, we have observed a 4.8% incidence of vs-CMO following DMEK, which is higher than our incidence of pseudophakic CMO but lower than our incidence of CMO after PKP (unpublished data). Our findings are in line with those of previous publications on EK and DMEK in particular (Table 3). In our study, we included eyes with comorbid conditions, which may be confounding factors in the limited postoperative visual acuity following DMEK. However, in these eyes CMO was considered as “visually significant” since there were not *de novo* changes in the preexisting comorbidities that would account for the “lower-than-expected postoperative visual acuity”, and especially since visual acuity improved after CMO treatment in these cases.In our study, we have found that 60% of eyes had subretinal fluid in addition to intraretinal fluid cysts, higher compared with the reported incidence in previous studies^19,20^; we have observed no cases of isolated SRF, in agreement with the study by Flanary et al.^5^. The pathophysiologic mechanisms underlying postoperative CMO after EK is similar to that of pseudophakic CMO, with the additional release of inflammatory mediators due to iris manipulation by iridectomy and intracameral gas tamponade; one study found that iris damage before and after DMEK increased the risk of post-DMEK CMO^9^, and preoperative LASER iridotomy may be associated with a lower risk of post-EK CMO^21^. In eyes that underwent DMEK, there is an increased concentration of pro-inflammatory mediators IFN-γ, IL-8, and IL-10 compared with eyes without signs of corneal disease scheduled for cataract surgery^22^, and an amplified innate immune response (increased IL-5 and IL-8 levels) may play a role in failing DMEK grafts^23^; this pro-inflammatory milieu may explain the increased risk of post-DMEK CMO compared with cataract surgery in eyes without corneal endothelial disease. We have not quantified objectively the postoperative flare, which is a potential limitation of our study; objective measurements of postoperative intraocular inflammation (e.g. laser flare-cell photometry) could possibly provide further evidence of the role of inflammation in the pathophysiology of macular edema following EK.

**Table 3:** Published results on the outcomes of postoperative cystoid macular oedema after endothelial keratoplasty.

Author [REF]	Journal	Year of Publication	Country	EK eyes (n)	Type of EK graft	CMO eyes (n)	Fuchs’ dystrophy in eyes with CMO (%)	Incidence of postoperative CMO	Average onset of CMO	Mean BCVA at diagnosis of CMO	Mean foveal thickness at diagnosis (µm)	Management of CMO	Additional treatment [Type, n]	Time to resolution of CMO	BCVA after treatment	Foveal thickness after treatment (µm)	Comments
Heinzelmann et al.^8^	Br J Iphthalmol	2015	Germany	155	DMEK	20	80.6%	13.00%	N/R		401	Topical prednisolone acetate 1%%	YES [Intravitreal injections anti-VEGF and triamcinolone, 1]	N/R			Eyes with shorter axial length had a higher risk of CMO following triple surgery; CMO after DMEK did not increase risk of post-DMEK CMO in the fellow eye
Hoerster et al.^19^	Am J Ophthalmol	2016	Germany	150	DMEK	9	98.0%	6.00%	5.2 ± 2.0 weeks	Mean BCVA decreased by 0.15 logMAR	507 ± 170 (100% had IRF / 22% had SRF)	Topical prednisolone acetate 1% q1H 1 week then tapered ± ACTZ 250 mg 2id 2wk in one patient	YES [intravitreal 0.7-mg dexamethasone implant, 1]	2.3 ± 1.2 months	Mean BCVA increased by 0.16 logMAR after initiation of therapy	349 ± 199	One eye had persitent CMO after 12 months despite Ozurdex treatment; Early intensified postoperative topical steroid therapy was effective prophylaxis for post-DMEK CMO
Flanary et al.^5^	Cornea	2016	USA	173	DMEK	13	93.1%	7.51% incidence of vs-CMO	All cases appeared within 1 month	N/R		Topical prednisolone acetate 1% 4id + Topical kerotolac 0.5% 4id	NO [0]	Within 6 months of treatment	All cases BCVA ≥ 20/30 and 69% ≥ 20/25	N/R	The incidence of vs-CMO after DMEK was similar in the setting of recent or remote cataract surgery
Quilendrino et al.^27^	Cornea	2017	The Netherlands	500	DMEK	5	89.2%	1.00%	N/R			Topical NSAID + Intensified topical CS ± ACTZ	YES [intravitreal injections anti-VEGF, 1]	N/R			80% of cases had staged surgery (cataract preceding DMEK up to 12 weeks)
Pedemonte-Sarrias et al.^26^	Int J Ophthalmol	2017	Spain	55	DSAEK	6	83.3%	10.91% incidence of vs-CMO	5.8 ± 4.3 weeks	N/R		Topical NSAID or Sub-Tenon CS ± Topical CS	YES [repeated sub-Tenon steroids, PPV, 1]	2.2 ± 1.7 months	N/R		One eye had persistent CMO; the incidence of CMO was higher with triple surgery compared with standalone DSAEK
Kocaba et al.^3^	Cornea	2018	France	74	DMEK	11	50.0%	14.86%	Most cases appeared between M1 and M3	0.30 logMAR	467	Topical indomethacine 0.1% 3id 2mo OR Topical dexamethasone/neomycin 3id 2mo + ACTZ 500 mg 3id	YES [Intravitreal 0.7-mg dexamethasone implant, 1]	N/R			Differences between standalone DMEK vs triple surgery not statistically significant
Kitazawa et al.^24^	Jpn J Ophthalmol	2018	Japan	334	DSAEK	18	55.6%	5.39%	All cases appeared within 1 month	N/R		Topical NSAIDs	NO [0]	1 month	0.39 logMAR	N/R	This group previously reported glaucoma-related corneal endothelial failure was a potential risk factor for post-DSAEK CMO [ref. 15]
Inoda et al.^9^	Cornea	2019	Japan	77	DMEK	12	32.0%	15.58%	All cases appeared within 1 month	N/R	542.4 ± 23.1	Topical bromfenac + Sub-Tenon injection triamcinolone acetonide	NO [0]	N/R	0.12 logMAR	N/R	Iris damage scores, air volume in the AC, simple DMEK and rebubbling were independent risk factors for post-DMEK CMO in Asian eyes
Ching et al.^6^	Curr Eye Res	2020	Canada	209	DMEK	8	92.8%	3.83% incidence of vs-CMO	8.9 ± 2.1 weeks	0.85 ± 0.52 logMAR	442 ± 149	Topical prednisolone acetate 1% + nepafenac	YES [Periocular triamcinolone, 2]	4.1 ± 1.7 months	0.17 ± 0.15 logMAR	274 ± 29	Differences between standalone DMEK vs triple surgery not statistically significant
Lohmann et al.^20^	Graefe Arch Clin Exp Ophthalmol	2021	Germany	107	DMEK	8	79.4%	7.48%	All cases appeared within 1 month	0.38 ± 0.92 logMAR	432.0 ± 97.6 (100% had IRF / 38% had SRF)	Topical nepafenac 1% 3id added to topical CS therapy until resolution, then tapered	NO [0]	N/R	0.14 ± 0.69 logMAR	297.5 ± 24.3	Rebubbling was significantly associated with CMO after uncomplicated DMEK in patients without systemic and surgery-related risk factors;
Myerscough et al.^4^	Br J Iphthalmol	2021	Italy	2233	DSAEK + DMEK	63	36.4%	2.82% incidence of vs-CMO (5.56% after DMEK / 2.36% after DSAEK)	4.3 ± 6.6 months	N/R		Topical Dexamethasone 0.1% 4id + Bromfenac 2id + ACTZ 500 mg 3id 1mo then taper 6mo	NO [0]	N/R			DMEK (OR 2.42), age > 67 years(OR 2.35), diabetes (OR 3.16) are independent risk factors for vs-CMO
Guindolet et al.^7^	Br J Iphthalmol	2021	France	246	DMEK	23	72.8%	9.36% incidence of vs-CMO	N/R	0.40 (0.16—0.52) logMAR	N/R	Topical keterolac and/or ACTZ 250 mg 3id in addition to topical CS	NO [0]	7 (4–11) months	0.15 (0.10—0.35) logMAR	N/R	History of ERM, PPBK, and intraoperative hyphaema were independent risk factors for vs-CMO; incidence of vs-CMO higher after standalone DMEK compared with triple surgery
Present study			Spain	291	DMEK	14	50.0%	4.8% incidence of vs-CMO	4 (3–10) weeks	0.60 (0.40–0.80) logMAR	582.5 (400.0–655.0)	Add Topical NSAID 2id + ACTZ 250 mg 3id to topical CS regime	YES [Intravitreal CS, 2]	3 (2–3.5) months	0.30 (0.15–0.40) logMAR (all cases ≥ 20/40, and 50% ≥ 20/20)	278.0 (258.0–294.0)	See “Results” and “Discussion” sections
											(100% IRF/60% SRF)						

*EK* endothelial keratoplasty, *CMO* cystoid macular oedema, *BCVA* best-corrected visual acuity, *AC* anterior chamber, *DMEK* Descemet’s membrane endothelial keratoplasty, *ACTZ* acetazolamide, *vs-CMO* visually significant cystoid macular oedema, *NSAID* non-steroidal anti-inflammatory drug, *CS* corticosteroid, *VEGF* vascular endothelial growth factor, *IRF* intraretinal fluid, *SRF* subretinal fluid, *DSAEK* Descemet stripping automated endothelial keratoplasty, *OR* odds-ratio; *ERM* epiretinal membrane, *PPBK* pseudophakic bullous keratopathy, *N/R* not reported.

Proposed strategies to decrease risk of post-EK CMO include: (1) recognition of preoperative risk factors for postoperative CMO; (2) possibly preoperative laser iridotomy^21^, (3) early intensified postoperative topical CS therapy^19^, and (4) possibly postoperative topical NSAIDs. Hoerster et al. compared two groups of DMEK patients who were treated with either topical CS 5 times daily vs topical CS hourly for the first postoperative week [19,] and found a 0% incidence of post-DMEK CMO in the hourly topical CS patient group, compared with a 12% incidence in the group receiving topical CS 5 times daily. Evidence for the potential benefit of postoperative NSAIDs comes from a previous study that found a protective effect of NSAID in preventing CMO after DSAEK^24^, from extrapolation for published evidence regarding the effectiveness of NSAID for the prevention of pseudophakic CMO^16,25^, and from indirect evidence by Inoda et al.^9^, who found an increased risk of CMO after standalone DMEK compared with staged DMEK surgery, hypothesized to be due to the post-cataract surgery treatment which included topical NSAIDs. Although we did not perform a statistical analysis to compare the risk of CMO between staged DMEK and standalone DMEK, in our case series most patients had undergone standalone DMEK surgery rather than staged surgery.

As mentioned, the first strategy to decrease the risk of post-EK CMO is recognition of preoperative risk factors. However, several studies have analyzed preoperative and perioperative risk factors for CMO after EK, with conflicting findings. In our study, there was a relatively high proportion of non-FECD eyes, including two redo DMEK eyes, suggesting that indications other than FECD may represent a risk factor for vs-CMO. In several studies reporting CMO after EK (but not all), a relatively high proportion of cases occurred in non-FECD eyes^4,9,24^, and previous studies have reported that glaucoma-related corneal endothelial dysfunction, and PPBK were risk factors for CMO after EK^7,17,18^; however, whether indication for DMEK is a risk factor for postoperative CMO remains unclear^3,4,9^.

In our study, three eyes had history of DMEK in the fellow eye, however we do not believe that DMEK in the fellow eye was a contributing factor for vs-CMO after DMEK in the second eye^4,8^. Two eyes needed rebubbling after DMEK; while it is conceivable that rebubbling may increase the risk of post-EK CMO due to repeated manipulation and resulting in increased release of prostaglandins and other inflammatory mediators^9,20,26^, this association has been disputed by other studies^3,4,6,8,19^.

A high proportion of eyes that developed vs-CMO following DMEK in our study had previous history of pseudophakic CMO in the operated eye or in the fellow eye. Previous studies have excluded eyes with previous history of CMO from analysis, as this has been considered an uncontrollable risk factor for postoperative CMO^19^; however, we opted to include them in our study, since the main objective of our study was to analyze the outcomes of eyes with post-DMEK vs-CMO.

Although the findings by Myerscough et al. suggest that DMEK is associated with a 2.4-fold possibility of vs-CMO compared with DSAEK^4^, we find it interesting to note that our incidence of vs-CMO after DMEK compares favorably with the reported incidence of vs-CMO following DSAEK in another study conducted in Spain^26^, despite the limitations inherent to such a comparison.

In our study, most cases of vs-CMO developed during the first postoperative month after DMEK, and all cases developed vs-CMO within 6 months after DMEK. Reported average times to detection of post-EK CMO range from 5.2 weeks and 4.3 months [4, 6, 19, 26], with most cases appearing within the first month of surgery^3,5,9,20,24^. A standardized management protocol for postoperative CMO following EK is currently lacking. Proposed first-line approaches have included increased frequency of topical CS, topical NSAIDs, oral acetazolamide, and sub-Tenon CS injection, alone or in combination (Table 3). Our standard first-line treatment for post-DMEK vs-CMO consists of adding a topical NSAID 2id plus oral acetazolamide 250 mg 3id to the postoperative topical CS regime; in line with the work by Hoerster et al. showing that intensified topical CS in the early postoperative period reduce the risk of CMO^19^, since two years we have also increased the frequency of topical CS during the first postoperative week to one drop every 2 hours, with close monitorization of IOP. This protocol appears to be an effective first-line approach for post-EK CMO, since most eyes achieved resolution of CMO within 2–6 months; second-line treatment consisting of intravitreal corticosteroid injection was successful in resolving CMO in those eyes with partial response to first-line treatment in the two cases we observed, although one of the eyes eventually had a complicated clinical course with rejection at M8 + IOL opacification requiring IOL exchange + anterior vitrectomy, and SGF at M21 post-DMEK. Median BCVA improved to 0.30 logMAR following resolution of CMO, and continuous improvement in BCVA was observed at last follow-up observation (median final BCVA 0.02 logMAR). In previous studies (Table 3), the prognosis of post-EK CMO was favorable in most patients, with resolution of macular edema and improvement in BCVA ranging between 0.12 and 0.39 logMAR; Hoerster et al. reported a mean 0.16 logMAR increase in BCVA after initiation of therapy^19^. In most published series, only very few cases required second-line treatments^3,6,8,19,26,27^, and only two case series have reported cases of persistent CMO after EK, in which the authors reported refractory CMO despite second- and third-line treatments^19,26^.The main limitation of our study is the presence ofcases of LTFU and cases of eyes where OCT data was not available in the patients’ EMRs, which is a limitation of study’s retrospective design. However, we highlight that the primary objectives of the study were to determine the proportion of eyes with vs-CMO after DMEK and the improvement in BCVA after treatment of vs-CMO, which are not affected by the missing data on CRT measurements.

In conclusion, this study reinforces the notion that postoperative vs-CMO is a relatively infrequent but potentially vision-threatening complication after DMEK surgery, and more frequent than pseudophakic CMO. Visual prognosis is good following treatment, with resolution of CMO with first-line treatment in a large proportion of cases. Although our study was not powered to determine risk factors for post-DMEK vs-CMO, we observed a high proportion of eyes that underwent DMEK for indications other than FECD, a high proportion of eyes with previous history of pseudophakic CMO, and that most eyes in our case series had undergone standalone DMEK rather than staged DMEK surgery. We believe that a standardized protocol for the management of post-EK CMO is needed, and future multicentric, prospective, randomized clinical trials are encouraged to ascertain the preoperative and perioperative risk factors for post-EK CMO, and to determine the most effective first- and second-line treatments for this postoperative complication.

## Supplementary Information

Supplementary Legends.

Supplementary Table 1.

## Data Availability

The datasets used and/or analyzed during the current study are available in the final version of the manuscript (Supplementary Table 1).
