# Supplementary material for: Outcomes of cystoid macular edema following Descemet’s membrane endothelial keratoplasty in a referral center for keratoplasty in Spain: retrospective study
Source: Sci Rep. 2023 Feb 9;13:2375. doi: 10.1038/s41598-023-29127-5 (PMC9911599; doi:10.1038/s41598-023-29127-5)
Supplement: Supplementary file 1 — Supplementary Legends. [file 41598_2023_29127_MOESM1_ESM.docx]

**Supplemental Table 1 –** Individual patient data, including baseline preoperative data and clinical outcomes.

*Legend: OS – left eye, OD – right eye; PPBK – pseudophakic bullous keratopathy; FECD – Fuchs’ endothelial corneal dystrophy; DMEK – Descemet membrane endothelial keratoplasty; IOL – intraocular lens; CMO – cystoid macular oedema; T2DM – type 2 diabetes mellitus; AC – anterior chamber; ARMD – age-related macular degeneration; NPDS – non-penetrating deep sclerectomy; BCVA – best-corrected visual acuity; AL – axial length; ACD – anterior chamber depth; ECC – endothelial cell count; CRT – central retinal thickness; IRF – intra-retinal fluid; SRF – sub-retinal fluid; vs-CMO – visually significant cystoid macular oedema; ACTZ – oral acetazolamide; IOP – intraocular pressure; F-U – follow-up time; MD – missing data; N/A – not applicable; LTFU – loss to follow-up.*
